# Shifts in COVID-19 mortality throughout pandemic: a five-year analysis of healthcare responses

**DOI:** 10.31744/einstein_journal/2025CE1751

**Published:** 2025-10-03

**Authors:** Camilla Mattiuzzi, Giuseppe Lippi

**Affiliations:** 1 Rovereto Hospital Provincial Agency for Social and Sanitary Services Trento Italy Medical Direction, Rovereto Hospital, Provincial Agency for Social and Sanitary Services, Trento, Italy.; 2 University of Verona Section of Clinical Biochemistry Verona Italy Section of Clinical Biochemistry, University of Verona, Verona, Italy.

Dear Editor,

The coronavirus disease 2019 (COVID-19) pandemic has unprecedentedly affected the global society, healthcare systems, and economies.^(1)^ Analyzing the place of death of patients with COVID-19 over the first five years of the pandemic offers valuable insights into shifting healthcare dynamics. Therefore, this study aimed to assess trends in COVID-19-caused patient deaths to better understand how healthcare and social infrastructure adapted to the evolving crisis.

We conducted an electronic search of the US National Center for Health Statistics (NCHS) WONDER (Wide-Ranging Online Data for Epidemiologic Research) Provisional Multiple Cause of Death online database.^(2)^ This search identified all death certificates listing COVID-19 (International Classification of Diseases, 10th Revision [ICD-10] code U07.1: "COVID-19 confirmed by laboratory testing, irrespective of severity") as a cause of death in the US between 2020 and 2024. The search results were grouped by year and place of death and reported as the total number of death certificates containing ICD-10 code U07.1. This study required no ethical approval because the NCHS WONDER is a publicly available, anonymized, and freely searchable database.

**Figure 1:**
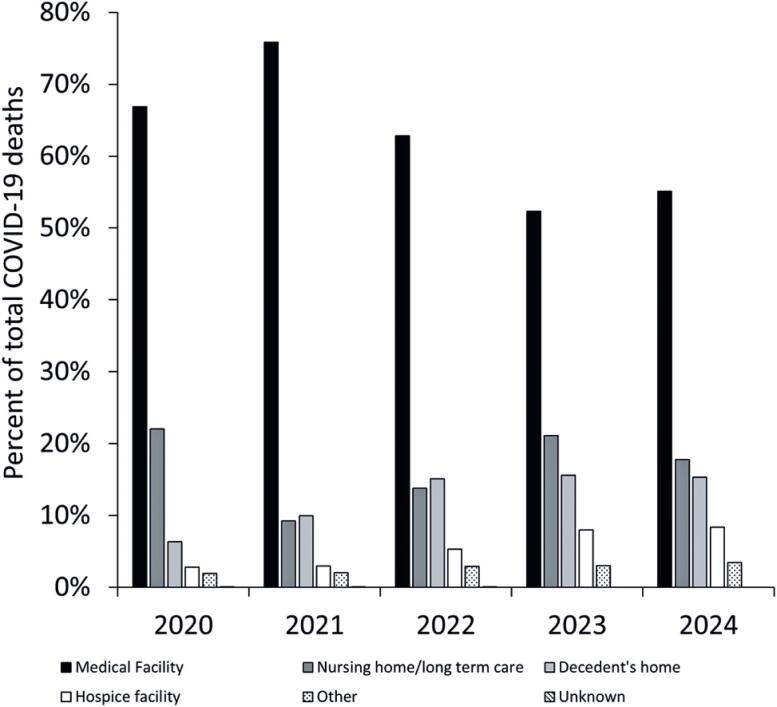
Distribution of COVID-19 deaths in the US based on the place of death during the first five years of the pandemic

The number of COVID-19 death certificates with the ICD-10 code U07.1 in the US increased from 388,577 in 2020 to 466,236 in 2021; however, then declined to 249,659 in 2022, 80,499 in 2023, and 51,645 in 2024. The results of our analysis are illustrated in figure 1. The proportion of deaths in medical facilities peaked in 2021, followed by a significant decline in the subsequent years. COVID-19 deaths in nursing homes and long-term care facilities dropped significantly between 2020 and 2021, followed by fluctuations, with a partial recovery in 2023 and slight decline in 2024. In contrast, the rate of COVID-19 deaths in hospice facilities steadily increased from 2020 to 2024. Similarly, the number of at-home COVID-19 deaths has consistently increased from 2020 to 2024. Deaths in other locations demonstrated a slight increase but remained below 3.5 % throughout the study period. Chi-square statistical analysis indicated a highly significant variation in the frequency distribution of COVID-19 deaths across different locations (X^2^=60,266; p<0.001).

The evolving pattern of COVID-19 deaths across locations reflects multiple factors, including the trajectory of the ongoing COVID-19 pandemic and healthcare system adaptations. The early years of the pandemic were characterized by a predominance of hospital deaths, likely owing to unprepared healthcare facilities, hospital overcrowding, lack of effective treatments, and severity of the initial outbreak, resulting in high hospitalization rates and subsequent in-hospital deaths.^(3)^ As the pandemic progressed, specifically from 2022 to 2024, hospitalization rates declined, thereby increasing the COVID-19-associated mortality in non-hospital settings. Fluctuations in nursing home deaths highlight the effects of policy interventions, infection control, and evolving treatments during the second and third waves of the pandemic. Efforts to prevent nursing home outbreaks in 2021 may have contributed to a temporary reduction in deaths in these settings.^(4)^ However, as restrictions eased and the virus continued to circulate, the proportion of nursing home deaths rebounded.

Notably, by 2024, 55.1 % of COVID-19 deaths occurred in hospital facilities, whereas 15.3 % occurred at home—a nearly 2.4-fold increase in at-home deaths since 2020. These findings highlight the need for continued investment in healthcare infrastructure, specifically home-based and hospice care, to better prepare for future public health crises.^(5)^
